# Complete Genome Analysis of a *Haemophilus parasuis* Serovar 12 Strain from China

**DOI:** 10.1371/journal.pone.0068350

**Published:** 2013-09-02

**Authors:** Yufeng Li, Amy H. Y. Kwok, Jingwei Jiang, Yao Zou, Fangyuan Zheng, Pan Chen, Chengcai Hou, Frederick C. Leung, Ping Jiang

**Affiliations:** 1 College of Veterinary Medicine, Nanjing Agricultural University, Nanjing, China; 2 Bioinformatics Center, Nanjing Agricultural University, Nanjing, China; 3 School of Biological Sciences, University of Hong Kong, Hong Kong SAR, China; Beijing Institute of Genomics, China

## Abstract

*Haemophilus parasuis* is the etiological agent of Glässer's disease in pigs and 15 standard serovars were identified. The widespread disease causes great economic loss in the swine industry worldwide. Aiming to investigate the differences in genome composition and functions among various strains, a highly virulent strain ZJ0906 of *H. parasuis* serovar 12 from China was analyzed and compared with serovar 5 SH0165. Strain ZJ0906 genome is 2,324,740 base pairs with 40.06% genomic GC content. It contains 2,484 open reading frames (ORF) predicted by Glimmer 3.02, of which 2,352 (∼94.7%) were annotated by NCBI nr blast, 1,745 by COG database and 1,829 by KEGG database. 109 potential virulence factors were annotated in strain ZJ0906 and 3 of which are potentially related to antibiotic resistance. Strain ZJ0906 genome is ∼55 kilobases longer than SH0165 genome, with an extra 211 predicted ORFs. VFDB, ARDB, and PAIDB blast searches showed that ZJ0906 and SH0165 shared a nearly identical panel of potential virulence factors, drug resistant genes and four PAI-like regions which showed high homology to *Enterococcus*, *Escherichia* and *Salmonella*. Synteny analysis showed that gene rearrangements are frequent between the two strains, which may lead to variations in pathogenicity and cross-protection among serovars. KEGG pathway analyses showed strain ZJ0906 shared similar metabolic pathways to strain SH0165. Molecular identification of these genomic elements and potential virulence factors pave the way to the better understanding of mechanisms underlying metabolic capabilities and pathogenicity of *H. parasuis* and prospective vaccine targets besides the widely used method of inactivated bacteria.

## Introduction

*Haemophilus parasuis* (*H. parasuis*) is an important respiratory-tract pathogen in pigs and the etiological agent of porcine polyserositis, polyarthritis and meningitis, known as Glässer's disease [1].To date, 15 *H. parasuis* serovars with apparent differences in virulence have been described [2]. However, a large number of non-typable isolates are frequently reported [3], [4]. Inconsistent cross-protection among serovars is one of the major problems for the control of Glässer's disease by means of bacterins [5]. It is essential to know the prevalent serovars in a given area to effectively control Glässer's disease, as vaccine immunity confers only limited cross-serovar protection [6]. Serovars 4 and 5 were the most prevalent serovars, followed by serovars 13, 14 and 12 in China [7], [8]. While in Brazil, serovar 4 was the most prevalent serovar, followed by serovars 5, 14, 13 and 2 [9]. Genomic characterization of *H. parasuis* serovar 5 was completed recently [10], [11], but the differences in genomic structure and composition among different serovars and whether these differences associated with pathogenesis and antigenic variations are not clear.

In China, Glässer's disease is common among finishing pigs and causes great economic loss. Many type strains have been isolated by our laboratory from pigs with clinical symptoms of high fever. In this study, the genome of the field strain ZJ0906 of *H. parasuis*, previously confirmed as serovar 12, was sequenced and compared with serovar 5, with focus on the investigation of potential virulence factors, drug resistant genes and pathogenicity-like islands in *H. parasuis*.

## Materials and Methods

### Bacterial strain and genome DNA extraction

The *H. parasuis* field strain serovar 12 ZJ0906 were cultured in tryptic soy agar (TSA) or tryptic soy broth (TSB; OXOID, Hampshire, ENGLAND) supplemented with 10 mg/ml nicotinamide adenine dinucleotide (NAD) and 5% bovine serum. Bacterial genomic DNA was purified with the QIAGEN Blood & Cell Culture DNA Kit, following manufacturer's instruction.

### Pyrosequencing and complete genome assembly of *H. parasuis* ZJ0906

To confirm the purity of the genomic DNA of *H. parasuis* ZJ0906, 16S rDNA-specific region was amplified and 20 individual positive clones were sequenced by Genetic Analyzer 3130 (Invitrogen, Grand Island, US). BLASTn analysis [12] revealed that rDNA sequences have high similarity to those from various serovars of *Haemophilus parasuis* publicly accessible. The quality and quantity of genomic DNA were evaluated by 0.7% agarose gel electrophoresis and Nanodrop2000 (Thermo Scientific, Waltham, US), and using the Quant-iT Picogreen dsDNA kit (Invitrogen), respectively.

A whole genome shotgun library was generated with 500 ng of ZJ0906 genomic DNA. The shotgun sequencing procedure was performed using 454 GS Junior General Library Preparation Kit, following the manufacturer's instruction (Roche, Basel, Switzerland). In addition, an 8 kb-span paired end library was generated with 15μg of genomic DNA. The paired end sequencing procedure was performed using 454 GS Junior Paired end Library Preparation Kit, following the manufacturer's instruction (Roche). Paired end reads were used as orientation guide for assembling the contigs into scaffolds. The DNA libraries were amplified by emPCR and sequenced by FLX Titanium sequencing chemistry (Roche). One shotgun run and one paired end run were performed on individual libraries prepared with the same genomic DNA sample. After sequencing, the raw data were assembled by Newbler 2.7 (Roche) with default parameters. Primer pairs were designed along the sequences flanking the gap regions for PCR gap filling. The complete genome was submitted to NCBI Genbank and is publicly accessible (accession no: CP005384).

### Genome annotation of *H. parasuis* ZJ0906

Glimmer 3.02 [13] was used for gene prediction in *H. parasuis* ZJ0906 complete genome. All predicted ORF sequences were translated into amino acid sequences by in-house Perl scripts. BLASTp [14] was applied to align the amino acid sequences against the NCBI non-redundant (nr) database (January, 2013). Amino acid sequences with alignment length over 90% of its own length and over 40% match identity were chosen and the description of the best hit (with highest alignment length percentage and match identity) was assigned as the annotation of predicted gene. Intergenic regions were annotated by RepeatMasker (http://www.repeatmasker.org) with default parameters.

### Phylogenetic analysis of *H. parasuis* ZJ0906

Complete genomes of 4 *Haemophilus spp*. including *H. parasuis* serovar 5 SH0165, *H. ducreyi* 35000HP, *H. somnus* 129PT, and *H. influenzae* Rd KW20 and 4 closely related bacteria from other Genus [11] – *Actinobacillus pleuropneumoniae* serovar 3 JL03, *Pasteurella multocida* Pm70, *Mannheimia succiniciproducens* MBEL55E and *Actinobacillus succinogenes* 130Z (Accession numbers: NC_011852, NC_002940, NC_008309, NC_000907, NC_010278, NC_002663, NC_006300, and NC_009655, respectively) – and the draft genome of *H. parasuis* serovar 5 29755 (Accession number: NZ_ABKM00000000) were downloaded from NCBI Genbank. Orthologous genes were identified by BLAT [15] using Glimmer-predicted *H. parasuis* ZL0906 genes as queries against each of the 8 complete, annotated genomes as database. Predicted genes of *H. parasuis* ZJ0906 which were found as a single copy, and with 90% minimum alignment length against the other 8 bacteria were designated as the core genes. All 26 core genes were then aligned by MUSCLE [16] and concatenated. A Bayesian phylogenetic tree was constructed using MrBayes [17] using the consequent concatenated genes as the dataset and GTR+G+I as the substitution model. The chain length was set to 10,000,000 (1 sample/1000 generations) whilst the burn-in was set as 2000 after checking on the trace files of two independent runs with Tracer v1.4 (http://tree.bio.ed.ac.uk/software/tracer/).

For comparison within the species of *H. parasuis*, reciprocal BLAT was performed between the 3 strains, and numbers of orthologs (single copies in each strain) shared between them were calculated by in-house Perl scripts.

### COG analysis of *H. parasuis* ZL0906

BLASTp [14] was applied to align the amino acid sequences against the COG database [18]. Amino acid sequences with alignment length over 90% of its own length and over 20% match identity were chosen and the description of the best hit (with highest alignment length percentage and match identity) was assigned as the annotation of predicted gene. All annotated genes were then classified based on their COG classes. COG-annotated genes of strain ZJ0906 were compared to that of strain SH0165.

### Virulence gene and pathogenicity island analysis of *H. parasuis* ZJ0906

BLASTp [19] was applied to align the amino acid sequences against the VFDB database [20]. Amino acid sequences with alignment length over 90% of its own length and over 20% match identity were chosen and the description of the best hit (with highest alignment length percentage and match identity) was assigned as the annotation of predicted gene.

Pathogenicity islands were annotated using PAI Finder (https://www.gem.re.kr/paidb/pai_finder.php?m=f) on PAIDB [21], after preprocessing of the predicted genes into 400-orf input files by in-house Perl scripts.

Strain ZJ0906 virulence factors and PAI-like genes were compared to that of strain SH0165.

### Drug resistant gene analysis of *H. parasuis* ZJ0906

BLASTp [12] was applied to align the amino acid sequences against the ARDB database [22]. Amino acid sequences with alignment length over 90% of its own length and over 40% match identity were chosen and the description of the best hit (with highest alignment length percentage and match identity) was assigned as the annotation of predicted gene. All annotated genes were designated by the antibiotics to which they render the bacteria resistance. Comparison of antibiotics resistance genes were carried out between strain ZJ0906 and the other 8 bacteria chosen.

### Potential horizontal transferring gene analysis of *H. parasuis* ZJ0906

BLASTp [12] was applied to align the amino acid sequences against the ACLAME database [23].Amino acid sequences with alignment length over 90% of its own length and over 40% match identity were chosen and the description of the best hit (with highest alignment length percentage and match identity) was assigned as the annotation of predicted gene. All annotated genes were classified according to their corresponding potential horizontal transferring vectors (“virus” or phages in bacteria, plasmid or prophage). Horizontal transferring genes of strain ZJ0906 were compared to that of strain SH0165.

### Pathway analysis of *H. parasuis* ZJ0906

Glimmer-predicted ORF sequences of strain ZJ0906 were translated into amino acid sequences by in-house Perl scripts. All sequences were submitted to KEGG database [24] for automatic pathway annotation (http://www.genome.jp/kaas-bin/kaas_main). All annotated pathways were manually downloaded and curated by in-house Perl scripts.

## Results and Discussion

### Complete genome sequencing and assembly of *H. parasuis* ZJ0906

*Haemophilus parasuis* ZJ0906 genome was sequenced and its complete *de novo* assembly was achieved by one shotgun run and one 8 kb-span paired end run via Roche GS Junior, and the follow-up PCR gap filling and Sanger sequencing. A total of 137,580 raw shotgun reads (59,446,391 bases) and 91,425 raw paired end reads (28,335,685 bases) were generated by respective pyrosequencing runs, in which ∼99.60% and ∼97.10%, respectively, were aligned into 258 contigs and 10 scaffolds, resulting in an average sequencing depth of ∼34-fold. The average read lengths for the shotgun and paired end run are ∼432 base pair (bp) and ∼310 bp, respectively. The size of the largest scaffold is 2,326,318 bp which contains 125 large contigs and the N50 contig is 32,107 bp long, suggesting that this raw assembly is highly continuous. The complete circular genome of *H. parasuis* ZJ0906 was found to be 2,324,740 bp in length, with genomic GC content of 40.06% after PCR gap-filling by Sanger sequencing, highly similar to the previously published complete genome of *H. parasuis* SH0165 [11].

### Genome annotation of *H. parasuis* ZJ0906

**Figure 1:**
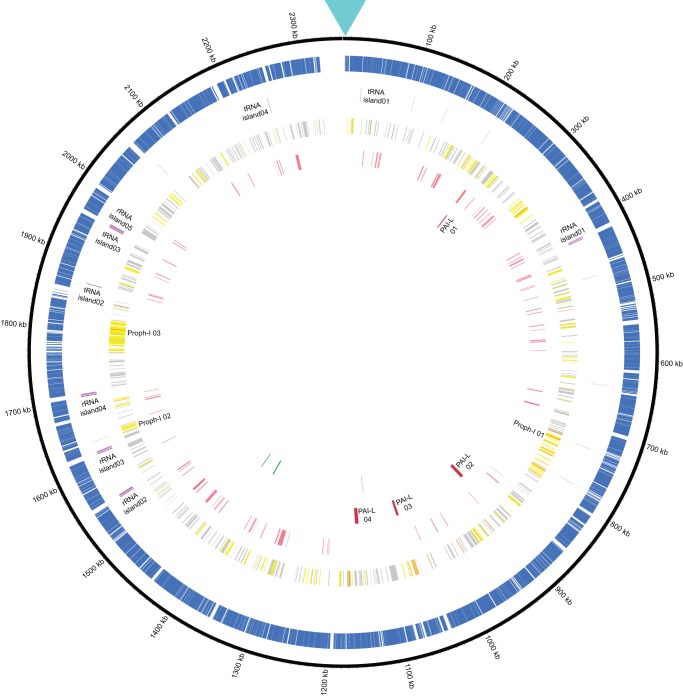
Circular representation of *H.* *parasuis* strain ZJ0906 genome. From the outer to inner layers, the circle shows (i) nucleotide positions in kilobases (kb) (black); (ii) CDSs annotated by NCBI non-redundant (nr) database (blue); (iii) RNA regions whereas rRNA islands and tRNA islands were labeled accordingly (purple); (iv) ACLAME database-annotated horizontal transferring genes, classified by their putative origins – plasmids (grey), prophages (yellow) and phages (orange) whereas the 3 major prophage islands were labeled accordingly; (v) VFDB-annotated potential virulence genes (light red); (vi) PAIDB-annotated PAI-like regions labeled accordingly (dark red); ARDB-annotated potential drug-resistance genes (dark green).

The *H. parasuis* ZJ0906 chromosome encodes 2,484 predicted genes (Glimmer 3.02), in which 2,325 (∼93.60%) was annotated by BLASTp search via NCBI non-redundant (*nr)* database (Table 1). The full annotation result was attached as File S1. 54 tRNA genes and 18 rRNA genes were found in *H. parasuis* ZJ0906 genome. Majority of them were arranged as large RNA islands – 5 rRNA islands (loci located on nucleotide positions of 419,486 to 423,366 bp, 1,550,077 to 1,554,037bp, 1,618,551 to 1,622,580bp, 1,702,915 to 1,706,912bp and 1,950,682 to 1,954,711bp respectively) and 4 tRNA islands (located on 25,369 to 25,537bp, 1,864,190 to 1,864,824bp, 1,950,075 to 1,953,830bp, and 2,245,963 to 2,246,216bp respectively) (Fig. 1). Strain ZJ0906 genome contains the same number of tRNA and rRNA genes as strain SH0165 genome. Full annotation of repetitive sequences, such as low-complexity repeats, interspersed repeats and RNA regions is attached as File S2.

**Table 1:** Summary of *H. parasuis* strains ZJ0906, SH0165 and 29755 genomes.

	*Haemophilus parasuis*					
	Serovar 12 strain ZJ0906		Serovar 5 strain SH0165		Serovar 5 strain 12755^#^	
**Total genome size**	2324740 bp		2269156 bp		∼2.22 Mb	
**GC level**	40.06%		40.00%		39.80%	
	**no.**		**no.**		**no.**	
**Predicted ORF**	2484		2223		2244	
**Annotated ORF**	**no.**	**% ˆ**	**no.**	**% ˆ**	**no.**	**% ˆ**
** against KEGG database**	1825	73.47	1814	81.60	1798	80.12
** against NCBI ** ***nr*** ** database**	2325	93.60	*N.A.*	*N.A.*	*N.A.*	*N.A.*
** against COG database**	1745	70.25	1534	84.05		0.00
** against VFDB**	109	4.39	106	5.81	108	4.81
** against ARDB**	3	0.12	3	0.16	2	0.09
** against ACLAME database**	615	24.76	510	27.95	539	24.02
** plasmids**	391	15.74	333	18.25	339	15.11
** phages**	31	1.25	24	1.32	35	1.56
** prophages**	193	7.77	153	8.38	165	7.35
** against PAIDB**	4	0.16	4	0.22	*N.A.*	*N.A.*
	**length**	%*	**length**	%*	**length**	%*
**Repetitive sequence**	13791 bp	0.59	13791 bp	0.59	*N.A.*	*N.A.*

Note: ^#^ denotes only draft genome was available for strain 29755; ˆ denotes percentage to number of total Glimmer-predicted ORF in *H. parasuis* strain ZJ0906; N.A. defines where values are non-applicable; *denotes percentage as to total genome length of respective strains.

### Phylogenomic and phylogenetic analysis of *H. parasuis* ZJ0906

**Figure 2:**
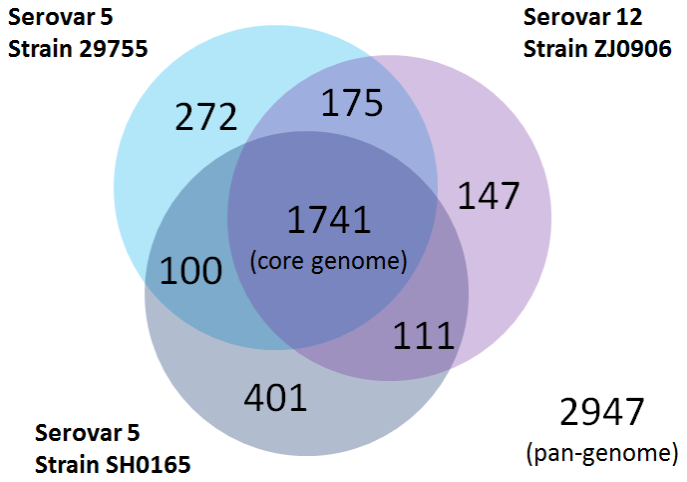
Pan-genome of *H.* *parasuis* strains ZJ0906, SH0165 and 29755. The Venn diagram was not drawn in proportion and aims only for illustration of pan-genome and distribution of core genes. Circles denote genomes, overlapping region between circles denote genes shared with respective genomes. Numeral figures within respective regions denote the number of genes found therein.

1,741 core genes (∼70.09% of predicted open reading frame (ORF) in strain ZJ0906) were identified between the two complete genomes of *H. parasuis* ZJ0906 and SH0165 and the draft genome of strain 29755. 1,916 potential orthologs (∼77.13%) were shared between *H. parasuis* strains ZJ0906 and 29755, which is a little more than that shared between strains ZJ0906 and SH0165 (1,852 orthologs), and between strains SH0165 and 29755 (1,841 orthologs) (Fig. 2). In the pan-genome (composed of 2,947 genes), 407 genes (∼13.61%) were only found in strain ZJ0906, while 147 genes (∼4.99%) and 272 genes (∼9.23%) were only found in strains SH0165 and 29755, respectively.

As shown in File S3, synteny between the two complete genomes of strains ZJ0906 and SH0165 is not very conserved. Chromosomal rearrangement was commonly observed along the whole stretch of the two genomes, especially a large proportion of the ∼1250–1425 kb region in strain SH0165 genome could not be matched to strain ZJ0906 genome (File S3).

On the other hand, amongst the other members within the family *Pasteurellaceae*, only modest numbers (<100) of orthologs to *H. parasuis* ZJ0906 were found, with the exception of *Actinobacillus pleuropneumoniae* serovar 3 JL03 which have 142 orthologs, even more than that found in species under the same Genus of *Haemophilus* (Table 2). In fact, only 45 genes are shared between the *Haemophilus* spp yet fully sequenced.

**Table 2:** Potential strain ZJ0906 orthologs found in complete genomes of other bacteria.

#	Name	NCBI Accession no.	No. of orthologs ˆ	% of CDS^#^
**1**	*Haemophilus ducreyi* 35000HP	NC_002940	89	3.58
**2**	*Haemophilus somnus* 129PT	NC_008309	69	2.78
**3**	*Haemophilus influenzae* Rd KW20	NC_000907	66	2.66
**4**	*Actinobacillus pleuropneumoniae*serovar 3 JL03	NC_010278	142	5.72
**5**	*Pasteurella multocida* Pm70	NC_002663	72	2.90
**6**	*Mannheimia succiniciproducens* MBEL55E	NC_006300	59	2.38
**7**	*Actinobacillus succinogenes* 130Z	NC_009655	58	2.34
	Shared between *Haemophilus spp.*		45	1.81
	Shared by all listed bacteria		26	1.05

Note: ˆ Orthologous genes were identified by BLAT [15] using Glimmer-predicted *H. parasuis* ZL0906 genes as queries against each of the 8 complete, annotated genomes as database with threshold of 90% alignment length and E-value equals 1e-05; ^#^defines percentage to number of total Glimmer-predicted ORF in *H. parasuis* strain ZJ0906.

**Figure 3:**
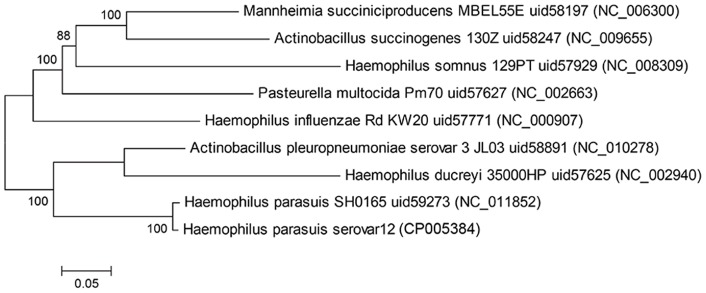
Bayesian phylogenetic tree of *H.* *parasuis* strain ZJ0906 and other closely related bacteria. Phylogenetic tree was constructed using MrBayes [17] using the random concatenation of 26 aligned core genes as the dataset and GTR+G+I as the substitution model. The chain length was set to 10,000,000 (1 sample/1000 generations) whilst the burn-in was set as 2000. Posterior probabilities are denoted at nodes.

The 26 core genes shared among *H. parasuis* ZJ0906 and the 8 closely related species with complete genomes were aligned and randomly concatenated before phylogenetic tree construction by MrBayes. Phylogenetic analysis showed that *H. parasuis* ZJ0906 shares the closest evolutionary origin to strain SH0165 as expected (Fig. 3). Interestingly, similar to number of orthologs (Table 2), *H. parasuis* ZJ0906 displays closer evolutionary relationship to *A. pleuropneumoniae* serovar 3 JL03 than to other *Haemophilus* species sequenced (Table 2), as previously mentioned for *H. parasuis* serovar 5 SH0165 by Xu's group [11]. Similar to *H. parasuis*, *A. pleuropneumoniae* is the etiological agent of a widespread severe pig disease, pleuropneumonia, which is commonly transmitted via airborne route or by direct contact. The close association between genes of the two species may be partially explained by the common habitat of *H. parasuis* and *A. pleuropneumoniae* in the upper respiratory tract of pigs. The co-habitat may have facilitated horizontal gene transfers between the two species.

### COG analysis of *H. parasuis* ZJ0906

**Figure 4:**
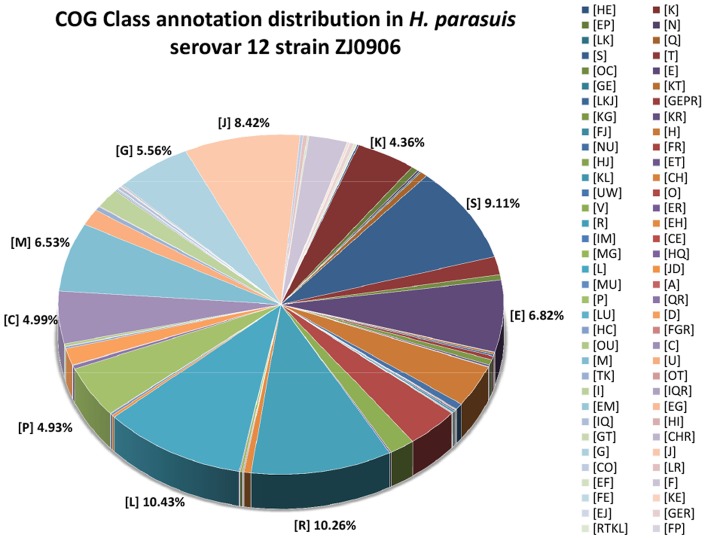
COG class distribution of *H.* *parasuis* ZJ0906 genome. The COG-annotated genes are grouped under their respective COG classes. Only their class abbreviations are used in this graph, their class descriptions are listed in Table 3. Percentages of the top ten classes are labeled for easy reference.

Orthologs are genes in different species that evolved from a common ancestral gene by speciation. Normally, orthologs retain the same function in the course of evolution. Thus, identification of orthologs is critical for reliable prediction of gene functions in a newly sequenced genome. NCBI COG database contains clusters of orthologous groups which provides genome-scale analysis of protein function prediction. 1,745 (∼70.25%) out of 2,484 Glimmer-predicted genes of *H. parasuis* ZJ0906 can be found in NCBI COG database (Table 1). COG-annotated class distribution for *H. parasuis* ZJ0906 was illustrated and the top 10 COG classes were annotated in Fig. 4. Majority of the genes, as expected, were involved in basic cellular functions, such as replication, transcription and metabolism, however, up to ∼19.68% of them only have predicted or unknown functions in COG database (Table 3). The single-letter COG class distribution of *H. parasuis* strains ZJ0906 and SH0165 was compared in Table 3. Differences in numbers of COG-annotated genes associated with the posttranslational modification, protein turnover, chaperones (COG class [O]), the defense mechanisms (COG class [V]), and the replication, recombination and repair (COG class [L]) were noted between the two strains. Difference in posttranslational modification and protein turnover may be associated with antigenic variations and cross protections across different strains, while discrepancy over the number of genes involved in defense mechanisms, DNA replication, recombination and repair may be attributed to their different adaptation to hosts. For the full COG functional annotation, please refer to File S4.

**Table 3:** Comparison of COG-annotated genes between *H. parasuis* strains ZJ0906 and SH0165.

	*H. parasuis* serovar 12 ZJ0906		*H. parasuis* serovar 5 SH0165		
COG_class	no. of ORF	%*	no. of ORF	%*	Description
[K]	76	4.355	70	4.563	Transcription
[N]	3	0.172	1	0.065	Cell motility
[Q]	9	0.516	7	0.456	Secondary metabolites biosynthesis, transport and catabolism
[S]	159	9.112	151	9.844	Function unknown
[T]	30	1.719	27	1.760	Signal transduction mechanisms
[E]	119	6.819	103	6.714	Amino acid transport and metabolism
[H]	70	4.011	67	4.368	Coenzyme transport and metabolism
**[O]**	**67**	**3.840**	**23**	**1.499**	**Posttranslational modification, protein turnover, chaperones**
**[V]**	**32**	**1.834**	**66**	**4.302**	**Defense mechanisms**
[R]	179	10.258	151	9.844	General function prediction only
**[L]**	**182**	**10.430**	**117**	**7.627**	**Replication, recombination and repair**
[A]	1	0.057	1	0.065	RNA processing and modification
[P]	86	4.928	71	4.628	Inorganic ion transport and metabolism
[D]	28	1.605	19	1.239	Cell cycle control, mitosis and meiosis
[C]	87	4.986	79	5.150	Energy production and conversion
[M]	114	6.533	105	6.845	Cell wall/membrane biogenesis
[U]	27	1.547	28	1.825	Intracellular trafficking and secretion
[I]	32	1.834	32	2.086	Lipid transport and metabolism
[G]	97	5.559	92	5.997	Carbohydrate transport and metabolism
[J]	147	8.424	145	9.452	Translation
[F]	49	2.808	44	2.868	Nucleotide transport and metabolism

Note: * Numbers of total COG-annotated genes in respective genomes were used as percentage bases. Significant differences in COG class distribution between *H.parasuis* strains ZJ0906 and SH0165 were highlighted in bold.

### Virulence gene and pathogenicity island analysis

Virulence genes of pathogenic bacteria, which code for toxins, adhesins, invasins or other virulence factors, may be located on transmissible genetic elements such as transposons, plasmids or bacteriophages [25]. Fifteen serovars of *H. parasuis* have been identified, serovars 1, 5, 10, and 12–14 may lead to the death of pigs and are considered to be highly virulent; serovars 2, 4, 8 and 15 are virulent, causing lesions in pigs, but serovars 3, 6, 7, 9 and 11 are considered to be avirulent [2]. To date, the relationship between serovars and virulence is not clear, especially the differences in mechanisms related to pathogenicity among highly virulent strains in *H. parasuis*, thus virulence genes and pathogenicity islands of strain ZJ0906 were analyzed and compared with strain SH0165.

**Figure 5:**
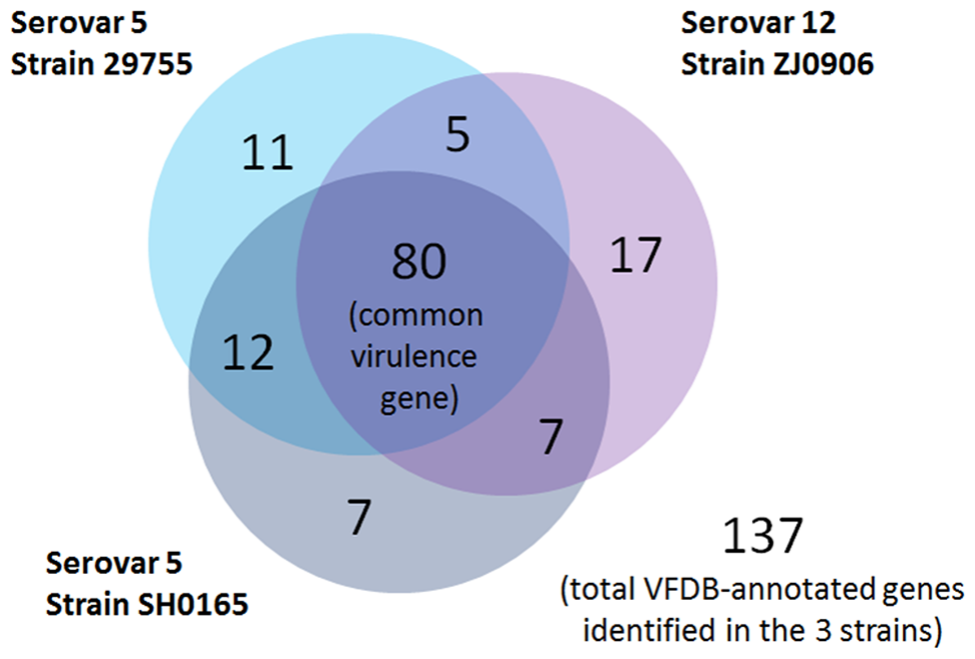
Common VFDB-annotated genes between *H.* *parasuis* strains ZJ0906, SH0165 and 29755. The Venn diagram was not drawn in proportion and aims only for illustration of the common VFDB-annotated genes shared between the 3 strains. Circles denote the panel of VFDB-annotated genes in the three strains, overlapping region between circles denote genes shared with respective strains. Numeral figures within respective regions denote the number of genes found therein.

109 potential virulence factors were annotated in strain ZJ0906 genome, while similar numbers (106 and 108) were identified in strains SH0165 and 29755 genomes, respectively. 80 of these VFDB-annotated genes were shared between the three strains, in which most of them are enzymes and transporter proteins involved in inorganic ion transport and acquisition, and lipopolysaccharide (LPS) biosynthesis (File S5). Among them, *RafD, Mip, galU, galE, rfaF, opsX* and *waaQ* were previously proved to be associated with adhesion and invasion of *H. parasuis* [26]–[28]. Among all 137 VFDB-annotated genes identified from the three strains, 92 (∼67.15%) were shared between strains SH0165 and 29755, while only 87 (∼63.50%) and 85 (∼62.04%) were shared between strains ZJ0906 and SH0165, and between strains ZJ0906 and 29755, respectively (Fig. 5). 17 potential virulence factors (∼12.41%) were found only in strain ZJ0906, while 7 and 11 were found only in strains SH0165 and 29755, respectively.

In addition to the VFDB search, a list of potential virulence factors previously mentioned in the literature was compiled and identified in strain ZJ0906 genome. These include genes involved in bacterial adherence such as the type IV fimbriae-like structure-encoding gene cluster, *pilA/B/C/D*, and *pilF*, and genes associated with surface LPS biosynthesis including the putative gene clusters related to major surface-exposed O-specific antigen biosynthesis previously hypothesized in SH0165 genome (Table 4).

**Table 4:** Identification of other potential virulence factors in *H. parasuis* ZJ0906 genome.

Locus tag	Gene	Functional description	Accession no.	%ID	COG class	COG no.
**Adhesion and secretion**						
contig00001_orf00032	*lspA*	lipoprotein signal peptidase	YP_002475845.1	99	[M][U]	COG0597
contig00001_orf00499	*secF*	preproteintranslocase subunit SecF	YP_002475539.1	99	[U]	COG0341
contig00001_orf00500	*secD*	preproteintranslocase subunit SecD	YP_002475540.1	99	[U]	COG0342
contig00001_orf00501	*yajC*	preproteintranslocase subunit YajC	YP_002475541.1	100	[U]	COG1862
contig00001_orf00655	*fimB*	fimbrial assembly chaperone	YP_002475354.1	98	[N][U]	COG3121
contig00001_orf00671	*aidA*	putative pertactin family virulence factor, outer membrane autotransporter/Type V secretory pathway, adhesinAidA	YP_002475345.1	81	*N.A.*	
contig00001_orf00862	*secG*	preproteintranslocase subunit SecG	YP_002475046.1	100	[U]	COG1314
contig00001_orf01225	*lolA*	outer-membrane lipoprotein carrier protein	YP_002474887.1	100	*N.A.*	
contig00001_orf01238	*nlpE*	lipoprotein copper homeostasis and adhesion, NlpE	YP_002474869.1	98	[M][P]	COG3015
contig00001_orf01241	*secA*	preproteintranslocase subunit SecA	YP_002474866.1	100	[U]	COG0653
contig00001_orf01489	*lepB*	signal peptidase I	YP_002474776.1	99	[U]	COG0681
contig00001_orf01723	*pulG*	Type II secretory pathway, pseudopilinPulG	YP_002476623.1	98	*N.A.*	
contig00001_orf01837	*pilF*	fimbrial biogenesis and twitching motility protein PilF-like protein	YP_002476534.1	100	[N][U]	COG3063
contig00001_orf01874	*yidC*	putative inner membrane protein translocase component YidC	YP_002475922.1	99	[U]	COG0706
contig00001_orf01875	*tatA*	twin arginine translocase protein A	YP_002475921.1	100	[U]	COG1826
contig00001_orf01876	*tatB*	sec-independent translocase	YP_002475920.1	99	[U]	COG1826
contig00001_orf01877	*tatC*	twin-arginine translocase subunit, sec-independent protein export TatC	YP_002475919.1	100	[U]	COG0805
contig00001_orf01888	*espP2*	putative extracellular serine protease (autotransporter)	YP_002475909.1	99	*N.A.*	
contig00001_orf01891	*espP1*	putative extracellular serine protease (autotransporter)	YP_002475906.1	99	*N.A.*	
contig00001_orf02030	*lolB*	outer membrane lipoprotein LolB	YP_002476445.1	97		
contig00001_orf02223	*secY*	preproteintranslocase subunit SecY	YP_002475951.1	100	[U]	COG0201
contig00001_orf02280	*pilA*	Tfppilus assembly protein, major pilinPilA	YP_002476429.1	99	[N][U]	COG4969
contig00001_orf02282	*pilB*	Tfppilus assembly pathway, ATPase PilB	YP_002476427.1	99	[N][U]	COG2804
contig00001_orf02284	*pilC*	Tfppilus assembly pathway, component PilC	YP_002476426.1	100	[N][U]	COG1459
contig00001_orf02285	*pilD*	Tfppilus assembly pathway, fimbrial leader peptidase PilD	YP_002476425.1	100	*N.A.*	
contig00001_orf02483	*ffh*	signal recognition particle GTPase	YP_002476013.1	99	[U]	COG0541
contig00001_orf02517	*secB*	preproteintranslocase subunit SecB	YP_002475987.1	100	[U]	COG1952
contig00001_orf02807	*secE*	preproteintranslocase subunit SecE	YP_002476272.1	99	*N.A.*	
contig00001_orf02829	*ftsY*	cell division protein, signal recognition particle GTPase	YP_002476303.1	95	[U]	COG0552
**LPS O-antigen biosynthesis**						
contig00001_orf01602	*neuA1*	CMP-N-acetylneuraminic acid synthetase	YP_002474693.1	98	[M]	COG1083
**contig00001_orf01601**	***wzx***	**putative lipooligosaccharideflippase**	**YP_002474694.1**	**95**	**[R]**	**COG2244**
**contig00001_orf01600**				**97**		
contig00001_orf01599	*lsgB*	CMP-N-acetylneuraminate-beta-galactosamide-alpha-2,3 -sialyltransferase/lipopolysaccharide biosynthesis protein	YP_002474695.1	99	*N.A.*	
contig00001_orf01598	*wzy*	putative O antigen polymerase	YP_002474696.1	99	*N.A.*	
contig00001_orf01597	*wcwK*	glycosyltransferase/capsular polysaccharide phosphotransferaseWcwK	YP_002474697.1	98	*N.A.*	
contig00001_orf01596 ˆ	*wcfQ*	extracellular polysaccharide glycosyltransferase	YP_002474698.1	99	[M]	COG0463
contig00001_orf01594 ˆ	*wbgX*	DegT/DnrJ/EryC1/StrS aminotransferase	YP_002474699.1	98	[M]	COG0399
contig00001_orf01593	*wbgY*	putative glycosyltransferase/lipopolysaccharide biosynthesis protein	YP_002474700.1	99	[M]	COG0399
contig00001_orf01592	*capD*	polysaccharide biosynthesis protein CapD	YP_002474701.1	99	[M][G]	COG1086
contig00001_orf01591	*wza*	polysaccharide export protein, periplasmic protein involved in capsular polysaccharide export	YP_002474702.1	99	[M]	COG1596
contig00001_orf01590	*ptp*	cytoplasmic tyrosine phosphatase	YP_002474703.1	100	[T]	COG0394
contig00001_orf01589	*wzz*	tyrosine kinase, chain length regulator in capsular polysaccharide biosynthesis	YP_002474704.1	99	[M]	COG3206

Note: ˆ contig00001_orf01595 is a pseudogene. Two adjoining copies of *wzx* genes were identified and highlighted in bold, possibly resulted from tandem duplication.

Pathogenicity-associated islands (PAIs) are distinct class of genomic islands where virulence genes have accumulated on the bacterial chromosome. PAIs, and their associated virulence genes, have spread among bacterial populations by horizontal gene transfer [25]. Four PAI-like regions were annotated by PAI finder (https://www.gem.re.kr/paidb/about_paidb.php?m=h) in strain ZJ0906 genome (Fig. 1), in which two of them contained considerable number of potential homologs of previously identified PAI-virulence genes (Table 5). In particular, homologs to all 4 putative virulence ORFs in *E. coli* pathogenicity island 1 (PAI 1) and *Salmonella* pathogenicity island 1 (SPI-1), which encode for potential chelated iron ABC transporter periplasmic-binding protein, chelated iron ABC transporter permease, chelated iron ABC transporter ATP-binding protein and ribosome-associated GTPase, were identified in PAI-L01 (Fig. 1 and Table 4). PAIs I to IV from *E.coli* strain 536 (I_536_ to IV_536_) encode a range of virulence factors, including P fimbriae, P-related fimbriae, α-hemolysin, S fimbriae, and the yersiniabactin siderophore system [29]. Similarly, as an indispensable virulence determinant, *Salmonella* pathogenicity island 1 (SPI-1) has gained much attention in host–pathogen interactions. It not only affects sophisticated activities during infection, including invasion, replication, and host responses, but also extends to other virulence-related aspects like biofilm formation [30]. The PAI 1/SPI-1-like region is also present in strain SH0165 genome, suggesting *H. parasuis* may manifest its pathogenicity by iron acquisition and persistent infection in hosts (Table 5). Since match identities of PAI-virulence genes to previously studied potential homologs only range from ∼44–77% (data not shown), further studies e.g. gene-knockout studies are necessary in elucidating the contribution of these PAI-like regions to pathogenicity in *H. parasuis*, especially in the investigation of correlation between the differences in PAIs and pathogenicity among highly virulent, virulent and avirulent strains of *H. parasuis*.

**Table 5:** Annotation of PAI-like regions in *H. parasuis* strain ZJ0906.

PAI-L region	Start	End	Size (bp)	No. of ORFs	No. of homologs of PAI- virulence genes	PAIs homologous to this region
PAI-L01	241604	243304	1701	4	4	Not named (*Enterococcus faecalis MMH594*)
						Not named (*Enterococcus faecalis V583*)
						PAI I 536 (*Escherichia coli 536*)
						SPI-1 (*Salmonella typhimurium LT2*)
						SPI-1 (*Salmonella enterica subsp. enterica serovar Typhi CT18*)
						SPI-1 (*Salmonella enterica subsp. enterica serovar Typhi Ty2*)
						SPI-1 (*Salmonella enterica subsp. enterica serovar Cholerae suis str. SC-B67*)
						Not named (*Salmonella typhimurium SL1344*)
PAI-L02	887763	894238	6476	9	0	YAPI (*Yersinia pseudotuberculosis 32777*)
PAI-L03	1054062	1058936	4875	10	0	SPI-7 (*Salmonella enterica subsp. enterica serovarTyphi CT18*)
						SPI-7 (*Salmonella enterica subsp. enterica serovarTyphi Ty2*)
PAI-L04	1143857	1151274	7418	9	2	Not named (*Enterococcus faecalis MMH594*)
						Not named (*Enterococcus faecalis V583*)

Details of the common virulence genes found between the 3 strains and information on annotated ORFs in PAI-L01 and PAI-L04 with potential homologs of virulence genes were included as File S6 and 7, respectively.

### Potential drug resistant gene annotation

**Figure 6:**
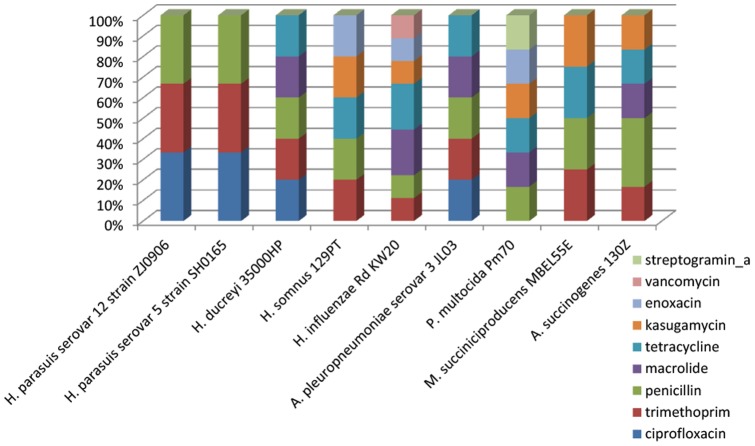
Comparative percentage of potential drug resistant genes. ARDB-annotated gene distribution of *H. parasuis* serovar 12 strain ZJ0906, serovar 5 strain SH0165, *H. ducreyi* 35000 HP, *H. somnus* 129PT, *H. influenzae* Rd KW20, *A. pleuropneumoniae* serovar 3 JL03, *P. multocida* Pm70, *M. succiniciproducens* MBEL55E and *A. succinogenes* 130Z were shown in percentage (in term of number).

Drug resistance is an evolutionary strategy in bacteria, which is often associated with horizontal gene transfer, such as plasmids and expression of some enzymes. *H. parasuis* is one of the most important swine pathogens, antimicrobial treatment is usually the most common, economical and effective means of disease control in husbandry. Although antimicrobial therapy is widely available for the prevention and control of clinical infections, the rising number of antibiotic resistant isolates is a growing global health concern for both human and animal populations [31]. Antimicrobial susceptibility studies of *H. parasuis* showed that most of them were resistant to penicillin, ciprofloxacin and trimethoprim+sulfamethoxazole [31], [32]. Our genomic analysis showed that 3 (0.12%) out of 2,484 Glimmer-predicted genes can be identified in ARDB database. Comparative study of *H. parasuis* strains ZJ0906 and SH0165 shows that both serovars have potential resistance against three kinds of antibiotics, i.e. ciprofloxacin, trimethopimand and penicillin (Table 6 and Figure 6). These results are in line with the previous experiments in *H. parasuis* species [31].

**Table 6:** Identification of drug resistant genes of *H. parasuis* strain ZJ0906 and comparison with strain SH0165.

	ARDB best blast hits					
Locus tag ˆ	Match ID (%)	Alignment length (bp)	Accession no.	Gene	Resistance to.	Orthologs in strain SH0165
contig00001_orf01386	44.85	165	CAL48457	dfra26	trimethoprim	YP_002475278.1
contig00001_orf01674	64.66	832	AAC22099	pbp1a	penicillin	YP_002476661.1
contig00001_orf01718	45.21	449	YP_001445425	norm	ciprofloxacin	YP_002476627.1

Note: ˆ denotes locus tag in strain ZJ0906.

Surprisingly, *A. pleuropneumoniae* has the same drug resistant genes with *H. ducreyi* and shares a similar panel (3 out of 5) of drug resistant genes with the two serovars of *H. parasuis*. This may be explained by the close ancestral origins between *A. pleuropneumoniae* and the 2 *Haemophilus* spp. – *H. ducreyi* and *H. parasuis*, as observed in our phylogenetic tree (Fig. 3). Given their common habitat in the pig respiratory tracts, horizontal gene transfer between *A. pleuropneumoniae* and *H. parasuis*, especially in pig farms where antibiotics are widely and commonly applied may act as a selective pressure for antibiotic tolerance strains and pose potential risks to global husbandry.

### Potential horizontal transferring genes analysis

It has been suggested that the integration of phage elements, as a strategy of horizontal gene transfer, play a potentially important role in genetic diversity and virulence variations in many bacteria [33]. The phage-related genes found in the *H. parasuis* ZJ0906 genome may also be a putative contributor to virulence and inheritance differences. For *H. parasuis* ZJ0906, 615 (24.76%) out of 2,484 Glimmer-predicted genes were annotated via *Blastp* search against the ACLAME database. Amongst these, 31 genes are potentially derived from phages, 391 genes from plasmids and 193 genes from prophages (Table 1), which are comparatively more than predicted in strain SH0165 genome. As previously noted in the synteny illustration (File S3), although the numbers were similar, the localization of prophage islands in the two genomes varies. Instead of an organization of three major islands (sizes over 9 kb) as described previously in strain SH0165 genome [11], phage-related genes are comparatively more scattered throughout strain ZJ0906 genome except from the three regions designated as the ∼5.8-kb Proph-I01 (from nucleotide positions of 728,879bp to 734, 713bp), ∼7.1-kb Proph-I02 (from 1,637,525bp to 1,644,584bp) and ∼47.77-kb Proph-I03 (from 1,768,270bp to 1,816,039bp). As strains ZJ0906 and SH0165 include ∼9.02% and ∼8.76% (of total Glimmer-predicted genes) of phage-related genes and some of the potential virulence factors annotated via VFDB database show overlapping with potential horizontal transferring genes (Fig. 1), implying that horizontal gene transfer may contribute to genetic variations and virulence variations among different strains.

### Pathway analysis of *H. parasuis* ZJ0906

KEGG-annotated genes found only in *H. parasuis* strains ZJ0906 and SH0165 respectively were listed in Table 7 and the full list of KEGG database-annotated genes were attached as File S8.

**Table 7:** KEGG-annotated genes not shared between *H. parasuis* strains ZJ0906 and SH0165.

Found in Strain ZJ0906 only		Found in Strain SH0165 only	
K00681	ggt; gamma-glutamyl transpeptidase [EC:2.3.2.2]	K00788	thiE; thiamine-phosphate pyrophosphorylase [EC:2.5.1.3]
K00928	lysC; aspartate kinase [EC:2.7.2.4]	K00851	E2.7.1.12; gluconokinase [EC:2.7.1.12]
K01002	E2.7.8.20; phosphoglycerol transferase [EC:2.7.8.20]	K00878	thiM; hydroxyethylthiazole kinase [EC:2.7.1.50]
K01011	TST; thiosulfate/3-mercaptopyruvate sulfur transferase [EC:2.8.1.1 2.8.1.2]	K00941	thiD; hydroxymethylpyrimidine/phosphomethyl pyrimidine kinase [EC:2.7.1.49 2.7.4.7]
K01079	serB; phosphoserine phosphatase [EC:3.1.3.3]	K01185	EARS; glutamyl-tRNAsynthetase [EC:6.1.1.17]
K01239	iunH; purine nucleosidase [EC:3.2.2.1]	K0371	fabK; enoyl-[acyl-carrier protein] reductase II [EC:1.3.1.-]
K01495	folE; GTP cyclohydrolase I [EC:3.5.4.16]	K02821	PTS-Ula-EIIA; PTS system, ascorbate-specific IIA component [EC:2.7.1.69]
K01572	E4.1.1.3B; oxaloacetate decarboxylase, beta subunit	K02840	waaB; UDP-D-galactose:(glucosyl)LPS alpha-1,6-D-galactosyltransferase [EC:2.4.1.-]
K01752	E4.3.1.17; L-serine dehydratase [EC:4.3.1.17]	K03080	L-ribulose-5-phosphate 4-epimerase [EC:5.1.3.4]
K12111	ebgA; evolved beta-galactosidase subunit alpha [EC:3.2.1.23]	K03277	waaU; heptosyltransferase IV [EC:2.4.-.-]
		K03475	PTS-Ula-EIIC; PTS system, ascorbate-specific IIC component

1,829 genes were annotated and the metabolic pathways including glycolysis and gluconeogenesis, the tricarboxylic acid (TCA) cycle and pentose phosphate pathway were analyzed. The general metabolic pathways identified in strain ZJ0906 were found to be highly similar to that in strain SH0165. As previously noted by Xu's group [11], the Entner-Doudoroff pathway was also not encoded in *H. parasuis* strain ZJ0906. *sgbE, sgbH* and *sgbU* genes encoded by strain SH0165 in the pentose and glucuronate inter-conversion pathway were found missing in strain ZJ0906 genome, which suggest that the inter-conversion of L-ribulose-5P, L-xylulose-5P and 3-dehydro-L-gulonate-6P is either absent, or a novel pathway or enzymes might be involved. Carbon source utilization is highly conserved between the two strains, except the identification of *ebgA* gene in strain ZJ0906 that may offer it an additional carbon source of lactose. Strain SH0165 genome contains ascorbate-specific PTS system IIA and IIC components, which were not identified in stain ZJ0906 genome. Since homologs to IIB component were not found in SH0165 genome, the functionality of this PTS in ascorbate metabolism remains questionable. Unlike strain SH0165, ORFs encoding two enzymes involved in pyruvate metabolism and amino acid metabolism – oxaloacetate decarboxylase, serine dehydratase – were found in strain ZJ0906, which enables important link formation between amino acid metabolism and pyruvate conversion, and subsequent TCA cycle and energy release. As a facultative anaerobe, it is not surprising that similar to strain SH0165, strain ZJ0906 also contains the *napF/D/A/G/H/B/C* operon encoding putative periplasmic nitrate reductase for anaerobic respiration. In addition, the same 3 two-component regulatory systems – *cpxA/R, arcA/B* and *qseB/C* – were annotated in strain ZJ0906, in which *arcA/B* genes potentially encode an anoxic redox control regulatory system. Likewise, heme biosynthetic pathway is fully conserved between the two strains while nicotinamide adenine dinucleotide (NAD) biosynthetic pathway is absent in both, as expected from their growth requirement with NAD (factor V) and without iron porphyrin (factor X) supplement.

Interestingly, gamma-glutamyltranspeptidase gene (*ggt*) involved in glutathione metabolism was only found in strain ZJ0906. This key enzyme was previously reported to act as an important regulator for intracellular homeostasis of oxidative stress, osmotic stress, and utilizing nutrients and facilitating growth in cysteine-limited habitats in other bacteria [34]–[36], hence its presence may influence capability of the strains in host survival. On the other hand, SH0165 genome encodes two enzymes involved in LPS biosynthesis that are absent in ZJ0906 – *waaB* and *waaU*. Previous studies have suggested minimal correlation between LPS and pathogenicity and host immunological responses in *H. parasuis* [6], yet LPS was shown to be an important contributor to pathogenicity as an endotoxin causing thrombosis in host blood circulation [37], hence difference in LPS may influence pathogenicity instead of protection against host.

## Conclusion

In present study, the complete genome of *Haemophilus parasuis* serotype 12 strain ZJ0906 from China, a highly virulent field strain of the etiological agent of swine Glässer's disease, was sequenced. The length of the genome is ∼2.3 million base pairs with genomic GC content of 40.06%. It contains 2,484 Glimmer-predicted ORF, of which 2,352 (∼94.7%) were annotated by NCBI nr blast, 1,745 by COG database and 1,829 by KEGG database. 109 potential virulence factors were annotated and 3 of which are potentially related to antibiotic resistance. This strain shared a nearly identical panel of potential virulence factors, drug resistant genes and PAI-like regions as serotype 5 strain SH0165. It was also found that gene rearrangements are frequent between the two strains, which may lead to variations in pathogenicity and cross-protection among serovars. These information should be useful to understand the mechanisms of the metabolic capabilities and pathogenicity of *H. parasuis* and develop a new vaccine to control this disease in the future.

## Supporting Information

File S1 **NCBI nr annotation for ZJ0906 genome.**
(XLSX)

File S2 **Repetitive sequence annotation for ZJ0906 genome.**
(XLSX)

File S3 **Synteny between** ***H. parasuis*** **strains ZJ0906 and SH0165.** The complete genome assemblies are depicted as thick black half-circle arcs, with their respective nucleotide positions marked in kilobases (kb). Brown ribbons joining the two arcs indicate conserved synteny. Chromosomal rearrangement was commonly observed in the two genomes, while a large proportion of the ∼1250–1425 kb region in strain SH0165 genome could not be matched to strain ZJ0906 genome.
(EPS)

File S4 **COG functional annotation for ZJ0906 genome.**
(XLSX)

File S5 **VFDB-annotated potential virulence genes in ZJ0906 genome.**
(XLSX)

File S6 **Common VFDB-annotated genes found between** ***H. parasuis*** **strains.**
(XLSX)

File S7 **PAIDB-annotated PAI-L01 and PAI-L04 in ZJ0906 genome.**
(XLSX)

File S8 **KEGG pathway annotation for ZJ0906 genome.**
(XLSX)
